# The Human Gene Mutation Database (HGMD^®^): optimizing its use in a clinical diagnostic or research setting

**DOI:** 10.1007/s00439-020-02199-3

**Published:** 2020-06-28

**Authors:** Peter D. Stenson, Matthew Mort, Edward V. Ball, Molly Chapman, Katy Evans, Luisa Azevedo, Matthew Hayden, Sally Heywood, David S. Millar, Andrew D. Phillips, David N. Cooper

**Affiliations:** 1 Institute of Medical Genetics, School of Medicine, Cardiff University, Heath Park, Cardiff, CF14 4XN UK; 2 i3S-Instituto de Investigação e Inovação em Saúde, Universidade do Porto, 4200-135 Porto, Portugal

## Abstract

The Human Gene Mutation Database (HGMD^®^) constitutes a comprehensive collection of published germline mutations in nuclear genes that are thought to underlie, or are closely associated with human inherited disease. At the time of writing (June 2020), the database contains in excess of 289,000 different gene lesions identified in over 11,100 genes manually curated from 72,987 articles published in over 3100 peer-reviewed journals. There are primarily two main groups of users who utilise HGMD on a regular basis; research scientists and clinical diagnosticians. This review aims to highlight how to make the most out of HGMD data in each setting.

## Introduction

The Human Gene Mutation Database (HGMD^®^) available via http://www.hgmd.org represents an attempt to systematically collate all known gene lesions underlying human inherited disease that have been published in the peer-reviewed literature. Mutation data catalogued by HGMD (summarized by mutation type) are listed in Table 1.

**Table 1:** Numbers and types of different variants and genes present in HGMD Professional release 2020.2 and the publicly available version of the database (as of June 7th 2020)

Mutation type	Number of Mutations in HGMD Professional 2020.2 (disease-associated/functional polymorphism sub-total)	Number of Mutations (publicly available via http://www.hgmd.org)
Missense substitutions	136,383 (6435)	85,225
Nonsense substitutions	31,407 (392)	20,779
Splicing substitutions (intronic and exonic)	24,976 (735)	17,183
Regulatory (5′ and 3′ and intergenic)	4723 (3006)	3544
Small deletions (≤ 20 bp)	41,749 (369)	28,155
Small insertions/duplications (≤ 20 bp)	17,760 (212)	11,745
Small indels (≤ 20 bp)	3813 (70)	2679
Gross deletions (> 20 bp)	20,448 (170)	14,186
Gross insertions/duplications (> 20 bp)	5219 (98)	3445
Complex rearrangements	2299 (138)	1747
Repeat variations	569 (331)	498
All HGMD data	289,346 (11,954)	189,186^a^
HGVS nomenclature provided^b^	263,452 (10,923)	0
Genomic coordinates/Variant Call Format (VCF) provided^c^	263,160 (10,845)	168,473^d^

Genes (subdivided by variant class)	Number of Genes in HGMD Professional 2020.2	Number of Genes (publicly available via http://www.hgmd.org)
Number of genes (with DM and/or DM? entries only)	7141	4218
Number of genes (with either DP, FP or DFP only)	1198	904
Number of genes (with a mixture of DM and/or DM? plus DP, FP and/or DFP)	2737	2522
Number of disease genes (containing at least one DM or DM? entry)	9878	6740
Total number of genes in HGMD^e^	11,076	7644

*DM* disease-causing mutation, *DM?* Likely disease-causing, but with questionable pathogenicity, *DP* disease-associated polymorphism, *DFP* disease-associated polymorphism with supporting functional evidence, *FP* in vitro/laboratory or in vivo functional polymorphism

^a^Mutations available via the HGMD Public Website (http://www.hgmd.org)

^b^As described in den Dunnen et al. (2016)

^c^As described by Danecek et al. (2011)

^d^The Ensembl HGMD_PUBLIC release (https://www.ensembl.org/) contains hg19/hg38 genomic coordinates and HGMD accession numbers only

^e^Total excludes mitochondrial genes (searchable but no variant data) and retired records

HGMD was originally established in 1996 with the goal of facilitating the scientific study of mutational mechanisms in human genes underlying inherited disease (Cooper et al. 2010; Stenson et al. 2017). However, over the last 20 years, it has acquired a much broader utility as it has become the central unified repository for disease-related genetic variation in the germ-line.

## Brief history of the resource

**Fig. 1:**
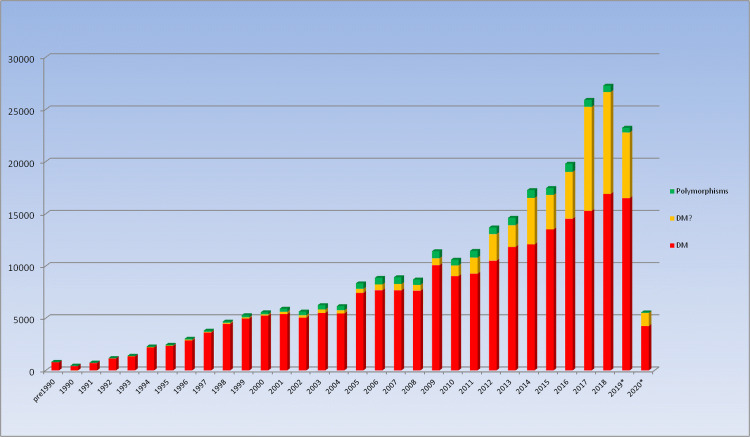
Mutation totals by year of publication subdivided by variant class. *Figures for 2019 and 2020 not yet complete. *DM* disease-causing mutation, *DM?* Likely disease-causing, but with questionable pathogenicity

The first Public version of HGMD containing ~ 10,000 variants in around 600 genes was made freely available from Cardiff via http://www.hgmd.org in April 1996. From that point, the database expanded swiftly to become the de facto central database for mutations causing human inherited disease. HGMD has been supported over the years by commercial partnerships with various industry leading biomedical research companies. Through a partnership with Celera Genomics from 2000 to 2005, HGMD data were made available as part of the Celera Discovery System. The years 2006–2015 saw the creation and continued development of HGMD Professional, a stand-alone web application, made available under license from BIOBASE GmbH. In 2015, QIAGEN Bioinformatics acquired BIOBASE, and our commercial partnership continued with HGMD data being made available via HGMD Professional (including data download) plus integration into Ingenuity Variant Analysis and Qiagen Clinical Insight. The latest version of HGMD (2020.2) contains 289,346 different mutations in 11,076 genes (Fig. 1).

## Sources of mutation data

HGMD screens the peer-reviewed biomedical literature on an ongoing basis, and currently contains data derived from over 72,000 manuscripts published in more than 3100 different journals. Relevant articles are identified via manual inspection of a core selection of journals, supplemented by the use of online computerised procedures utilising Google Scholar, publishers’ websites and PubMed, to survey the wider literature. Articles identified as potential sources of mutation data are assessed by a team of experienced curators (with an average of more than 12 years experience in curation). Discrepancies in variant reporting that require additional scrutiny are identified in approximately 20% of articles. Some 25% of these can be resolved by utilising other information reported in the manuscript or by referring to supplementary material (chromosomal coordinate, sequence chromatogram etc.). However, approximately 75% of these ambiguities necessitate direct contact with the authors. Author responses that are sufficient to allow us to include the mutation data in question are received for ~ 55% of queries; however, the reported variants from the other 45% (comprising ~ 7% of all papers screened) remain unresolved.

**Fig. 2:**
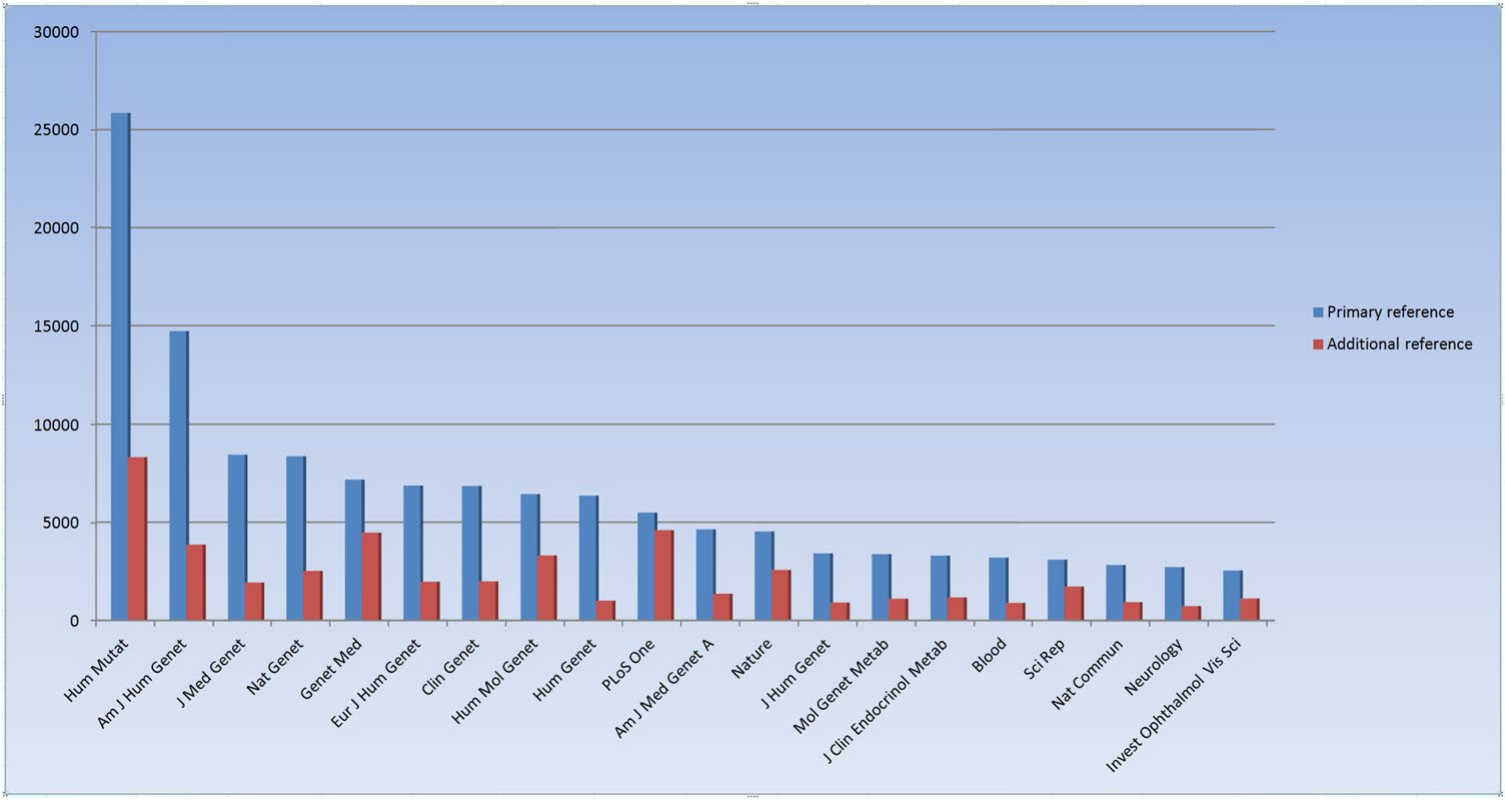
Top 20 journals by number of mutation entries (HGMD Professional release 2020.2 June 7th 2020) in relation to both primary and additional (secondary) references

One other challenge we have encountered is that an increasing number of journals do not appear to be systematically indexed by Medline, at least not immediately upon publication (i.e. the NLM catalogue states that the journal is not currently indexed for MEDLINE, although individually submitted abstracts may still be present in PubMed); with 729 mutation entries, the journal *Front Genet* is the most highly represented of these, followed by *Neurol Genet* (488 entries) and *Mol Syndromol* (333 entries). There are a total of 172 journals listed in HGMD that the NLM catalogue lists as not currently indexed by Medline/PubMed. This number represents approximately 5% of all the journals currently cited by HGMD. A summary of the top 20 journals cited in HGMD (by number of mutation entries listed) is shown in Fig. 2.

## Utilisation

There are many different ways in which HGMD data may be utilised in both a research and a clinical setting, all of which are dependent upon on the version of HGMD available to the user. For checking known genotype–phenotype relationships (i.e. relatively small numbers of variants found in specific genes involved in a known disease), the Professional (https://www.qiagenbioinformatics.com/products/human-gene-mutation-database/) online-only interface will most likely suffice. Users may search using the gene symbol, disease name utilising the Universal Medical Language System or UMLS (https://www.nlm.nih.gov/research/umls/), literature reference, HGVS description or genomic coordinate. More recently, nucleotide-level annotations for multiple non-canonical mRNA transcripts have been added to HGMD Professional, in recognition of the fact that clinically relevant variants are more likely to impact those exons that are present in multiple transcripts (Subramanian 2018).

Academic or non-profit users without a subscription may utilise the public version of HGMD (http://www.hgmd.org). However, this version is provided in a basic form that is searchable only by gene symbol or disease name, is only updated twice annually, is maintained permanently at least 3 years out of date, and does not contain any of the additional annotations or extra features present in HGMD Professional (e.g. dbSNP ID, chromosomal coordinates, HGVS nomenclature, Variant Call Format (VCF), population frequency data, additional literature reports, advanced search features, evolutionary conservation data and functional predictions).

**Fig. 3:**
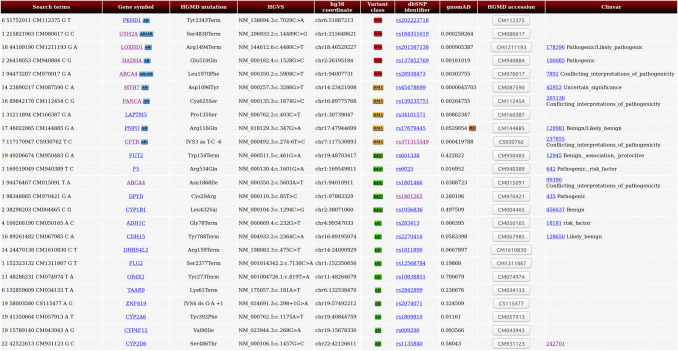
Example, online batch result set from HGMD Professional 2020.2

Increasingly in clinical practice (as has happened in research), the use of next-generation sequencing (NGS) technology has greatly expanded in recent years. HGMD has adapted to these changes by providing mutation data in standardized formats for ease of computational analysis. The most useful of these in a high-throughput context is Variant Call Format or VCF (Danecek et al. 2011), which mimics the format of the data that will typically be produced by a bioinformatician after processing the output from NGS. This format is available as part of the licensed HGMD Professional download version. There is also a publicly available dataset containing coordinates (but not precise nucleotide changes) released via Ensembl (https://www.ensembl.org). Users looking for a potential clinical diagnosis may wish to utilise the HGMD online batch search mode (utilising dbSNP identifier, hg19/hg38 coordinate, hg19/hg38 VCF, HGMD accession number, PubMed ID, HUGO gene symbol, HUGO gene ID, Entrez gene ID or OMIM ID). Results are limited to the first 500 found; however, these may be prioritised based on likely causation or population frequency based on gnomAD (http://gnomad.broadinstitute.org/) data. Results may also be restricted to a particular UMLS disease concept (see Fig. 3). In this way, Next Generation Sequencing results may be directly compared to HGMD data, and any relevant variants that have been previously implicated in disease causation are returned. Filtering a typical exome will generally yield a list of approximately 400 damaging variants, comprising up to 8 highly damaging DMs (Xue et al. 2012) and up to 15 potential risk alleles (Tabor et al. 2014). To make sense of these data, the user must prioritize their results in line with their own particular needs and priorities (Table 2).

**Table 2:** HGMD variant classes

HGMD variant class	Relevance	Clinical diagnostic setting	NGS research setting
DM—Disease-causing mutation	Literature indicates causal (or likely causal) link with disease	Most important. These data should be prioritized	Depending on the user’s remit, these should be looked at first
DM?—Likely disease-causing, but with additional uncertainty	As for DM, but the authors, curators or other literature evidence indicate that further caution is warranted	If no DM variants are found, these should be looked for next, or they should be ranked lower priority if there are DM results	These data may also be of interest, depending upon requirements (e.g. gene ontology or disease concept stratification)
DP—Disease-associated polymorphism	Significant statistical association with a clinical phenotype. Likely to be functionally relevant	Likely to be irrelevant in a clinical diagnostic setting	These should be included if personal disease risk is being assessed
DFP—DP with supporting functional evidence	As for DP, but definitive functional evidence exists (e.g. via an in vitro luciferase assay)	Potentially important in terms of calculating disease risk (e.g. venous thrombosis risk and Factor 5 Leiden). Other relevant disease risk or drug response variants are also present in this class	If the aim were to look at personal disease risk, or for disease modifiers, then these should be included
FP—functional polymorphism with no reported disease association	Functional effect has been demonstrated, but no disease association has been reported as yet	Likely to be irrelevant in a clinical diagnostic setting, although drug response variants may be present	Interesting from a research perspective as potential risk modifier variants
R—retired from HGMD	Record has been retired and is no longer considered to be phenotypically relevant	Potentially relevant for the purpose of variant exclusion	Potentially relevant if the researcher is interested in variant re-annotation etc

An exome screen will most likely return a combination of the different HGMD variant classes listed in Table 2. Results may be further prioritized within a variant class by (i) utilizing the population frequency data from dbNSFP3, a database of functional predictions and annotations based on genomic location (Liu et al. 2016), present in the HGMD download and (ii) making use of the HGMD computed ranking score. This ranking score is a single relative probability score between 0 and 1, with 1 being most likely disease-causing; it is currently available only for coding region nucleotide substitutions (both missense and nonsense). The score is computed by HGMD using a supervised machine learning approach known as Random Forest (Breiman 2001), and is based upon multiple lines of evidence, including HGMD literature support for pathogenicity (placed on a scale of 1–10, with 1 being the lowest score and 10 being the highest), evolutionary conservation (100-way vertebrate alignment), variant allele frequency and in silico pathogenicity prediction including CADD (Rentzsch et al. 2019), PolyPhen2 (Adzhubei et al. 2010) and MutPred (Li et al. 2009). HGMD data are used to train the model with disease-causing mutations (DM) forming the positive class and possible/probable disease-causing mutations (DM?) making up the negative class. Individually, ranking scores may be interpreted as relative probabilities of pathogenicity (i.e. the higher the score, the more likely the variant is to be disease-causing). Ranking scores may also be utilised in aggregate to prioritize and rank multiple HGMD variants that have been found in the same sample. A representative example result set (utilizing VCF search terms) from the HGMD Professional batch search showing the first five results from each mutation class present in a normal exome from an apparently healthy individual and sub-ranked by the HGMD ranking score, is provided in Fig. 3.

The top five results shown in Fig. 3 are all DM entries present in HGMD. The five DMs on the list are known to cause autosomal recessive disorders (*USH2A*, *PKHD1, LOXHD1*, *HADHA* and *ABCA4*), and the healthy individual concerned is, therefore, an asymptomatic carrier of these variants. However, *USH2A* and *ABCA4* have also been implicated in late-onset dominant phenotypes, and so may be of long-term clinical interest. The next five results in our illustrative list are from the DM? class and should therefore be treated with an additional degree of caution. However, upon closer inspection, some of these entries could prove to be of clinical relevance in the longer term. The *MYH7* variant is an autosomal dominantly inherited potential cardiomyopathy risk factor, whereas the *PNPO* variant may also be clinically relevant in relation to the phenotype of Pyridoxamine 5′-phosphate oxidase deficiency (although this phenotype can be highly variable). The remaining DM? entries listed in Fig. 3 display either recessive or more complex inheritance, or else give rise to biochemical phenotypes that are of relatively minor clinical concern, which is often the case for this class of variant.

The next three classes of variant (DFP, DP, FP) all occur at polymorphic frequencies, and therefore may confer increased disease risk, and/or may give rise to an alteration in the function of the gene/gene products involved. They are not, however, generally expected to be of immediate clinical concern. That said, HGMD would nevertheless recommend that DFPs, in particular the alleles with a low population frequency for the minor allele (Kido et al. 2018), should be treated as “honorary DMs” for the purposes of returning results, particularly in light of the problems that are often encountered when attempting to classify low-penetrance/hypomorphic alleles, or those with combinatory effects (Wang and Chiang 2019). Notable examples of clinically significant DFPs present in HGMD include the *DPYD* allele p.Cys29Arg (global MAF 0.28) which may be relevant for 5-fluorouracil toxicity and *F5* p.Arg534Gln (F5 Leiden – global MAF 0.02), both of which are listed in Fig. 3. Other selected examples of clinically important DFPs include *PROS1* p.Ser501Pro (Protein S Heerlen polymorphism – global MAF 0.002), *CD36* p.Tyr325* (global MAF 0.03), *AMPD1* p.Gln45* (global MAF 0.10), *CES1* p.Gly143Glu (global MAF 0.01), *FCN3* c.349delC (global MAF 0.02), *ABCA4* p.Asn1868Ile (global MAF 0.04). Some of the returned alleles in the DFP, DP and FP classes may also be relevant to drug metabolism or as potential modifiers of the clinical phenotype [for example the *SCN5A* p.His558Arg (global MAF 0.25) or *CYP2C19* c.681G > A *2 allele (global MAF 0.17)].

Population frequency data are often employed to screen out potentially benign alleles (Whiffin et al. 2017). Indeed, the HGMD curators have periodically utilised this method to re-annotate or remove questionable variants from HGMD. This practice should, however, in the opinion of the HGMD curators, be utilised with great caution, as it may reduce or even prevent the return of potentially clinically relevant alleles for certain later-onset diseases (Zernant et al. 2017; Wang and Chiang 2019). Despite these concerns, filtering by population frequency remains the best first method to de-prioritize low risk alleles from result sets. HGMD has therefore included population frequency data from 1000 Genomes (1000 Genomes Project Consortium et al. 2015), ExAC (Lek et al. 2016) and gnomAD (http://gnomad.broadinstitute.org/) to facilitate this process. FINDbase may also be used for specific populations (Kounelis et al. 2020). As a precaution, and owing to the fact that some disease-causing alleles occur at relatively high frequencies in certain populations, users may wish to consider “positive filtering”, and add HGMD-flagged alleles back into their result set if it is felt that they have been inadvertently excluded [e.g. *HFE* p.Cys282Tyr (MAF 0.038), *LPA* c.4289 + 1G > A (MAF 0.029), *HBB* p.Glu7Val (MAF 0.012) or *G6PD* p.Val68Met (MAF 0.033)].

## Further filtering

**Fig. 4:**
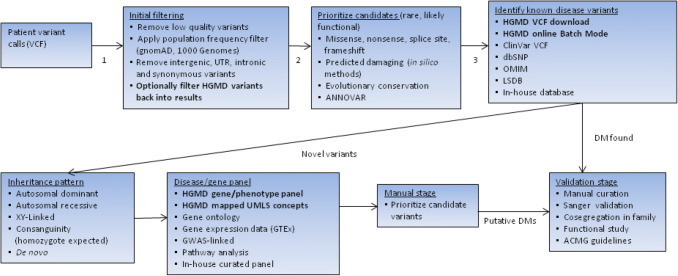
Example of an NGS/diagnostic workflow

The simplest way to filter variants (apart from by population frequency) is using in silico pathogenicity prediction methods. HGMD contains many of the predictions provided by dbNSFP3 (Liu et al. 2016), which may be utilised for this purpose. HGMD also contains terms present in the Gene Ontology database (The Gene Ontology Consortium 2017). This information (e.g. “mismatch repair”, “ATP binding” etc.) may be utilised if the user is aware of which ontology terms may be linked to their phenotype(s) or gene(s) of interest. Mapped UMLS disease concepts may be utilised to stratify results according to a broad set of disease-related terms (such as “Blood Disorder”, “Cancer” etc.). Where the phenotype is known, this approach is very efficient at identifying and returning HGMD alleles most relevant to the phenotype of interest, a method that is increasingly being recognized as important (Amin and Wilde 2018). An example of an exome prioritization/filtering workflow is given in Fig. 4.

## De novo mutations

Human germline de novo mutations are both a driver of evolution and an important cause of genetic disease (Goldmann et al. 2019; Veltman and Brunner 2012; Acuna-Hidalgo et al. 2016). Indeed, whole-genome studies have suggested that de novo mutations may be responsible for a considerable proportion of congenital or early-onset neurodevelopmental disorders, including autism spectrum disorder, epilepsy and intellectual disability/developmental delay (Neale et al. 2012; Iossifov et al. 2014; Hamdan et al. 2017). Although such disorders often display a complex multifactorial aetiology (Guo et al. 2018), it is thought that autism spectrum disorder in particular has a large risk of recurrence in families (Breuss et al. 2020). Although these studies are still at an early stage, they generally show a measurable effect on disease risk, especially for exonic loss-of-function (LOF) de novo variation (Takata 2019). HGMD has, therefore, taken the decision to include these mutations, owing to the increased likelihood of their being involved in de novo (non-familial) phenotypes.

De novo mutations identified as part of large-scale mutation screening programs in patients with developmental disorders are entered into HGMD under the DM? variant class unless there is convincing additional evidence to support their inclusion as DMs. All likely disruptive sequence changes identified in cases (but not controls, or unaffected siblings in parent–offspring groups) are entered. Such variants include single base substitutions causing missense, nonsense or canonical splice site changes as well as both small and large exonic frameshift deletions/insertions or other complex rearrangements. Other variant types (e.g. synonymous substitutions) may be considered for inclusion if additional evidence supportive of pathogenicity has been presented. This collection of de novo variants should prove useful to those undertaking large-scale screening programs in terms of checking for the known or suspected involvement of a particular gene or specific mutation (or mutation type) in a given neurodevelopmental disorder. Additional references will be added in the case of those mutations found recurrently in the literature, thereby adding to the weight of evidence supporting the involvement of a specific mutation or gene in a given disorder. There are now approximately 15,000 de novo mutations logged in HGMD; 2500 of these have at least one additional reference and 33 of them have been reclassified as DMs.

## Other resources

**Fig. 5:**
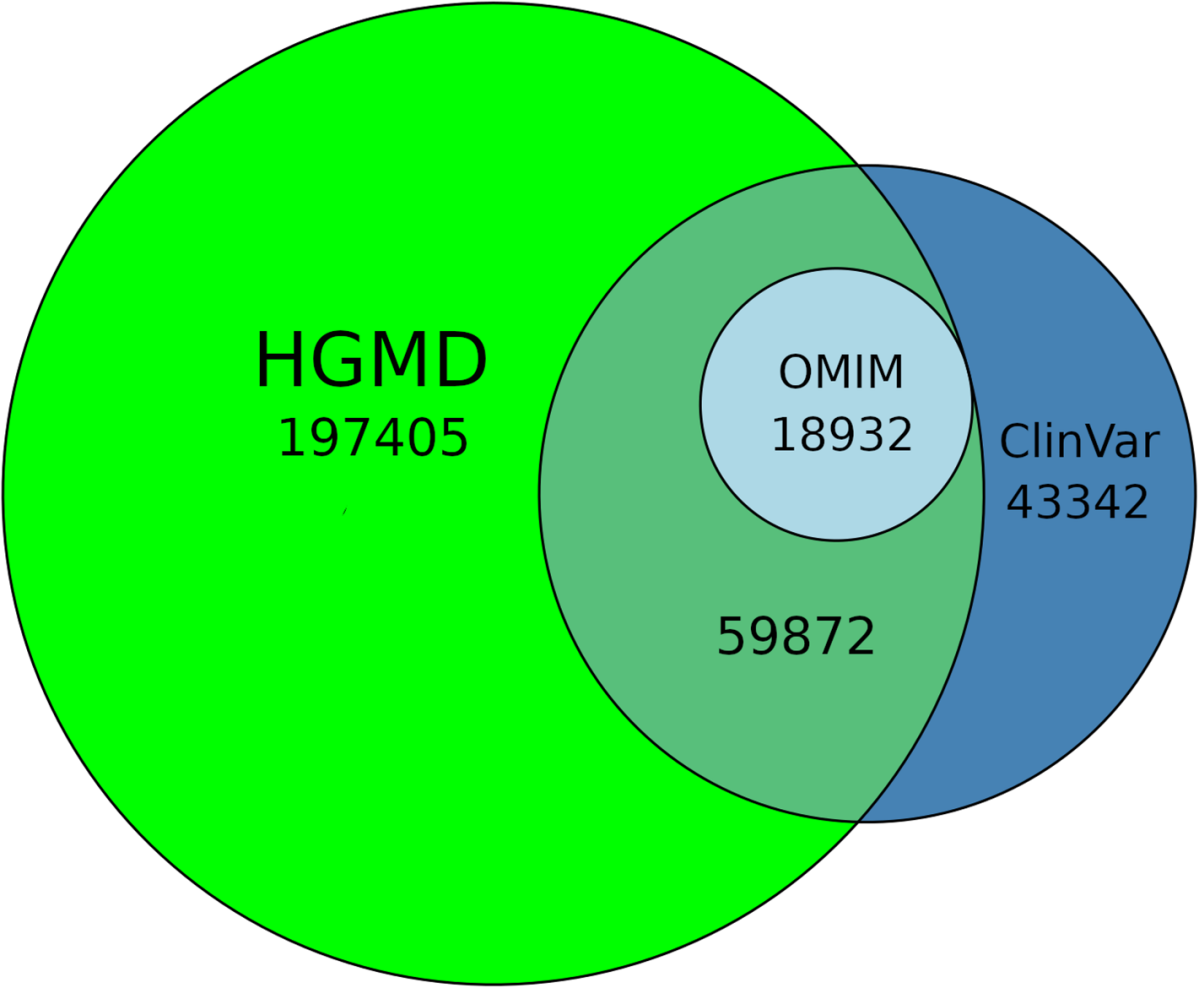
HGMD vs ClinVar vs OMIM comparison (as of March 2020)

There are a relatively small number of other sources (both publicly funded and commercial) of mutation data analogous to HGMD that are available to the scientific community. These include ClinVar (Landrum et al. 2020) (public), CentoGene (https://www.centogene.com) (commercial), LOVD (Fokkema et al. 2011) (software is public), COSMIC (Tate et al. 2019) (public and commercial license), DECIPHER (Bragin et al. 2014) (public), dbSNP (https://www.ncbi.nlm.nih.gov/snp) (public) and OMIM (Amberger et al. 2019) (public and commercial license). Like-for-like comparisons between these resources are very difficult, as obtaining the data can be problematic due to licensing requirements (CentoGene), or being distributed over many installations with potentially different usage/permission terms (LOVD). Although HGMD links to COSMIC, the data present in the latter resource are somatic in nature, and therefore, not directly comparable to HGMD. DECIPHER records data that are complementary to HGMD (i.e. large chromosomal rearrangements involving multiple genes which HGMD does not systematically catalogue). A basic comparison between the number of variants annotated by HGMD, ClinVar and OMIM is presented in Fig. 5.

Data for this comparison were limited to HGMD DM or DM? entries with genomic coordinates (March 2020 release 2020.1 VCF file) versus those ClinVar variants labelled with Pathogenic, Likely_pathogenic or Pathogenic/Likely_pathogenic assertions from the ClinVar VCF file (downloaded 2020-02-03). OMIM data were identified via the OMIM allelic variant identifier present in the ClinVar download. It can be seen that HGMD captures all OMIM pathogenic allelic variants, plus almost 60% of those present in ClinVar. In contrast, ClinVar captures only 23% of HGMD variants, with the majority (77%) remaining specific to HGMD. The remaining 43,342 ClinVar-only entries appear to comprise unpublished variants submitted by diagnostic laboratories (which complement HGMD’s attempts to provide comprehensive cover of the peer-reviewed literature). There is in addition a small overlap of ClinVar variants of uncertain significance/VUS (17,166 variants—not shown in Fig. 5) between HGMD and ClinVar. The bulk of ClinVar VUS (204,337 entries) are however not present in HGMD. The reason for this lies with HGMD editorial policy. Our curators will include a published VUS (as a DM?) if it is deemed plausible that the variant is the cause of the patient’s phenotype (e.g. VUS present in a known disease gene where it is rare in the general population, labelled VUS by the authors and is considered to be a reasonable potentially causative finding). ClinVar, however, appears to include practically all submitted VUS, even when the affected status of the individual or family is unknown, leading to much larger numbers of this type of variant being present in their dataset.

## Variant reclassification

HGMD will reclassify or retire a variant if published evidence comes to light (e.g. via functional, case-level or mass exome variant frequency studies) that supports reclassification. Variants may be reclassified from DM? to DM if the new evidence increases support for the potential pathogenicity of the variant in question. The reclassification can of course go the other way (DM to DM?) where new data emerge that argue against variant pathogenicity. Disease-associated polymorphisms (DP) may be reclassified to DFP if new evidence supporting a functional effect is published. A variant may also be retired (R) if found to have been included erroneously ab initio, or has subsequently been shown beyond reasonable doubt to be benign (either by virtue of its apparent population frequency, or literature reclassification or retraction). ClinVar variant reclassification rates (where a submitting laboratory updates a variant classification) are broadly similar to those of HGMD. The ClinVar reclassification rate has been reported to be 0.79% (Harrison and Rehm 2019), whereas the equivalent rate for HGMD data was 1.12% over the same time period (all data entered into HGMD between January 2016 and July 2019). However, if all HGMD entries are included (irrespective of the date when they were first entered), then the HGMD reclassification rate rises to 2.06%. This is to be expected, as HGMD has pursued a policy of continuous curation, re-annotating older data wherever necessary, whereas ClinVar appears to rely almost exclusively on the original submitter updating their submission (hence the lower rate of reclassification). Recent literature suggests that if ClinVar alleles are independently re-interpreted, then a large number of reclassifications become necessary (Xiang et al. 2020). Our view is that reclassification is to be expected with any well maintained mutation database, which should always be considered to be “work in progress”.

## Automated mutation retrieval

Researchers at HGMD have been involved for several years in attempts to automatically extract mutation data from the literature. We recently contributed towards the Automatic Variant Evidence Database (AVADA), a novel machine learning tool that uses natural language processing to automatically identify pathogenic genetic variant evidence in full-text primary literature (Birgmeier et al. 2020). AVADA automatically retrieved 58% of the likely disease-causing variants deposited in HGMD. Automatic retrieval and verification of novel likely disease-causing variants involved in inherited disease (i.e. those not already verified via manual curation) is, however, much more challenging. Our own internal assessment has demonstrated that > 90% of novel (i.e. unknown to HGMD) computationally derived automated literature mutation “hits” are in fact (from HGMD’s point of view) false positives comprising a mixture of several different types; (i) somatic mutations (e.g. cancer driver mutations or mutations conferring cancer therapy resistance), i(i) non-human mutations (i.e. from mouse, or another model organism), iii) artificially engineered mutations (e.g. mutagenesis experiments looking at catalytic or other active sites of a protein), (iv) so-called “A.I. artifacts” (i.e. supposed variant matches a specified text mining pattern, but is not a genuine mutation), (v) incorrectly or inadequately described human mutations (e.g. protein description not matching nucleotide description, requiring manual verification, but entered verbatim by the algorithm), (vi) secondary mRNA or other post-transcriptional sequelae (e.g. skipped exons or intron inclusions), (vii) coincidentally co-located mutations matching positions in two or more different genes, but only being genuine for one of them, and finally (viii) benign variants (polymorphisms or rare variants only found in healthy controls). The tracking down of such false leads involved a great deal of HGMD editorial/curation time, but did not lead to a corresponding increase in identified novel disease-relevant mutations. Owing to these limitations, we have opted to implement a strictly controlled form of automated additional reference retrieval, using pre-existing and well-described human-curated HGMD variants.

## Automated additional reference retrieval

To minimise the possibility of accruing the aforementioned false positives, HGMD has taken the decision to strictly limit the application of automated mutation retrieval/identification methods to our previously catalogued human mutation data set, identifying only the additional literature mentions of these already verified mutations. The computerised method employed will examine the full-text of identified articles (HTML or PDF including any supplementary material). Any literature reference in which a mention of a previously described mutation is found will be recorded and entered into HGMD as an additional reference for that mutation. These additional auto-curated references will be clearly marked as non-human curated when presented to HGMD users.

## Future plans

HGMD plans to include the data from the GTEx project (GTEx Consortium 2013) to allow filtering on tissue expression of particular genes. This, in combination with the Gene Ontology and UMLS, should allow even more efficient filtering (e.g. combining “central nervous system disorder” from the UMLS with “brain expressed” genes from GTEx). We also have plans to expand our provision of in silico variant predictions and to include computed ACMG classifications, based on the ACMG 2.0 rules recently published (Kalia et al. 2017). Roll-out of our automated additional reference retrieval system is also a priority.

## Conclusion

In conclusion, HGMD contains an expansive set of tools that may be utilised by users in the fields of clinical diagnostics, personalised genomics and NGS/bioinformatics research to search and prioritise results derived from its comprehensive mutation data set. The onus is, however, on the clinician or researcher to use these tools and data sensibly and appropriately to obtain results that are suitable for their own use cases.
